# The Carcinogenic Properties of Overlooked yet Prevalent Polycyclic Aromatic Hydrocarbons in Human Lung Epithelial Cells

**DOI:** 10.3390/toxics10010028

**Published:** 2022-01-09

**Authors:** Alison K. Bauer, Katelyn J. Siegrist, Melanie Wolff, Lindsey Nield, Thomas Brüning, Brad L. Upham, Heiko U. Käfferlein, Sabine Plöttner

**Affiliations:** 1 Department of Environmental and Occupational Health, Colorado School of Public Health, University of Colorado Anschutz Medical Campus, Aurora, CO 80045, USA; katelyn.siegrist@viatris.com (K.J.S.); lindseynield@alumni.mines.edu (L.N.); 2 Institute for Prevention and Occupational Medicine of the German Social Accident Insurance, Institute of the Ruhr-University Bochum (IPA), 44789 Bochum, Germany; wolff@ipa-dguv.de (M.W.); bruening@ipa-dguv.de (T.B.); kaefferlein@ipa-dguv.de (H.U.K.); 3 Department of Pediatrics and Human Development, Michigan State University, East Lansing, MI 48824, USA; upham@msu.edu

**Keywords:** polycyclic aromatic hydrocarbons, gap junctions, micronuclei, benzo[*a*]pyrene, fluoranthene, 1-methylanthracene, co-carcinogenic, lung, epithelial cells

## Abstract

The WHO classified air pollution as a human lung carcinogen and polycyclic aromatic hydrocarbons (PAHs) are components of both indoor (e.g., tobacco smoke and cookstoves) and outdoor (e.g., wildfires and industrial and vehicle emissions) air pollution, thus a human health concern. However, few studies have evaluated the adverse effects of low molecular weight (LMW) PAHs, the most abundant PAHs in the environment. We hypothesized that LMW PAHs combined with the carcinogenic PAH benzo[*a*]pyrene (B[*a*]P) act as co-carcinogens in human lung epithelial cell lines (BEAS-2B and A549). Therefore, in this paper, we evaluate several endpoints, such as micronuclei, gap junctional intercellular communication (GJIC) activity, cell cycle analysis, *anti*-BPDE-DNA adduct formation, and cytotoxicity after mixed exposures of LMW PAHs with B[*a*]P. The individual PAH doses used for each endpoint did not elicit cytotoxicity nor cell death and were relevant to human exposures. The addition of a binary mixture of LMW PAHs (fluoranthene and 1-methylanthracene) to B[*a*]P treated cells resulted in significant increases in micronuclei formation, dysregulation of GJIC, and changes in cell cycle as compared to cells treated with either B[*a*]P or the binary mixture alone. In addition, *anti*-BPDE-DNA adducts were significantly increased in human lung cells treated with B[*a*]P combined with the binary mixture of LMW PAHs as compared to cells treated with B[*a*]P alone, further supporting the increased co-carcinogenic potential by LMW PAHs. Collectively, these novel studies using LMW PAHs provide evidence of adverse pulmonary effects that should warrant further investigation.

## 1. Introduction

Polycyclic aromatic hydrocarbons (PAHs) are a prevalent class of toxicants that are found in indoor and outdoor sources around the world [1]. PAHs are of environmental and occupational concern for detrimental effects on human health. Due to strong epidemiological evidence, the International Agency for Research on Cancer (IARC) considers exposures to PAHs to be major risk factors for the development of lung cancer as well as other pulmonary diseases [2,3]. Environmentally, these toxicants are found as components of airborne particulate matter (PM, both PM_2.5_ and PM_10_) [1,4,5,6], gasoline and diesel exhaust [7,8,9,10], coal gasification, coke oven emissions, forest fire smoke, and firsthand, secondhand and thirdhand cigarette and marijuana smoke [5,11]. PAHs are also more prevalent in urban areas compared to rural settings, although with the onset of rapid climate changes due to increases in wildfires that is likely to change [1,4,5,6]. PAHs are also a concern for numerous occupations, such as mining, oil production, asphalt application, casino settings, among others [12,13,14]. There are more than 100 different PAHs identified in the environment [15], yet many of the PAHs have not been assessed for adverse health effects. Thus, there is a need for additional studies to determine PAH toxicities.

The reference PAH for toxicity for all PAHs is benzo[*a*]pyrene (B[*a*]P) that is an IARC group 1 carcinogen [16]. B[*a*]P has a ≥5 ring structure, is considered a high molecular weight (HMW) PAH (>207 g/mol) and is one of numerous HMW PAHs found in the environment. Other HMW PAHs are classified in the group 2A (*probably carcinogenic*) or 2B (*possibly carcinogenic*) categories [17]. There are 16 U.S. E.P.A. priority PAHs based on high levels of exposure to humans, 8 of which are considered low molecular weight (LMW) PAHs [18,19]. We define these LMW PAHs as non-genotoxic (except for naphthalene) and <206 g/mol, [17,19]. IARC classifies these overlooked and abundant LMW PAHs as group 3, “*not classifiable as to their carcinogenicity to humans*” [17,19], except naphthalene, a group 2B carcinogen. However, our past and current studies provide evidence that these LMW PAHs need to be re-evaluated for co-carcinogenic effects [20,21,22,23]. Additionally, these LMW PAHs (e.g., phenanthrene, anthracene and fluoranthene) are more prevalent than the HMW counterparts in indoor air pollution (e.g., firsthand, secondhand and thirdhand smoke), and outdoor air pollution [24,25]. Lastly, these LMW PAHs are components of food, sediment (particularly methlyanthracenes and methylphenanthrenes) and occupational exposures, such as asphalt, coal, coke and steel industry workers [1,17,26,27,28,29,30]. For example, dermal levels of 2–4 ring PAHs in asphalt workers averaged >60 μg/day [31].

We previously published evidence that, when two LMW PAHs (1-methylanthracene and fluoranthene) were combined with B[*a*]P in a mouse alveolar type II cell line (C10 cells), significant co-carcinogenic properties were observed [20]. The endpoints evaluated were gap junctional intercellular communication (GJIC), DNA adduct formation and an inflammatory mediator (cyclooxygenase 2, (COX2). Cell-to-cell communication via gap junctions is implicated in early stage carcinogenesis and is supported by considerable evidence in numerous cancers [32,33,34,35,36]. A stronger correlation existed for tumorigenicity for GJIC, an epigenetic event, than for mutagenicity when 251 chemicals were tested [36]. In addition, the Halifax Project consensus is that inhibition of GJIC is one of the hallmarks of cancer [37]. GJIC inhibition allows initiated cells to evade growth suppression along with other critical events of cancer, such as the suppression of p53/pRB pathway, and loss of contact inhibition, all of which allow cancer cells to escape from the normal feedback growth control mechanisms of a tissue [37]. The addition of a binary mixture of LMW PAHs containing a 1:1 mixture of 1-methylanthracene (1-MeA) and fluoranthene (Flthn) to B[*a*]P-treated cells significantly increased the GJIC-inhibitory effects as compared to B[*a*]P or the binary mixture alone. [20] The addition of this binary mixture of PAHs to B[*a*]P-treated C10 lung cells also exponentially increased benzo[*a*]pyrene diol-epoxide (BPDE)-DNA adduct and COX-2 mRNA levels [20].

In this paper, we provide further evidence for the co-carcinogenic properties of a binary mixture of LMW PAHs when combined with B[*a*]P in a human bronchial epithelial cell line (BEAS-2B) and in a human tumorigenic alveolar type II cell line (A549) at non-cytotoxic doses. Collectively, we hypothesize that LMW PAHs combined with the carcinogenic PAH benzo[*a*]pyrene (B[*a*]P) act as co-carcinogens in human lung epithelial cell lines (BEAS-2B and A549). We evaluate multiple endpoints to identify additional disease biomarkers, specifically cancer, that may initiate a more comprehensive risk assessment and policy changes in how LMW PAHs are regulated. The endpoints used are cytotoxicity, cell death, cell cycle analysis as an indicator of DNA replication during the S-phase and ultimately cell growth, micronuclei analysis and the formation of specific DNA adducts of B[*a*]P as measures of genotoxicity, gap junctional intercellular communication as a measure of interrupted tissue homeostasis, and cytochrome p450 1B1 (CYP1B1) immunoblots as an indicator of PAH mediated phase 1 enzyme induction. The novelty of this study is the demonstration that the non-genotoxic LMW PAHs, an overlooked class of PAHs in cancer risk assessments, increased the carcinogenic potential of the known carcinogenic PAH, B[*a*]P, and also induced cancer relevant effects in the absence of B[*a*]P. For this purpose, we use 1-methylanthracene and fluoranthene, a binary PAH mixture that has been previously used and represents two PAHs common in all forms of cigarette smoke, PM exposures including those of wildfires, and in sediments [5,21,30].

## 2. Materials and Methods

### 2.1. Chemicals

For the experiments performed in the United States, fluoranthene (Flthn; purity 97.2%) was purchased from AccuStandard (New Haven, CT, USA), benzo[*a*]pyrene (B[*a*]P; purity ≥ 96%), dimethyl sulfoxide (DMSO) and Lucifer yellow from Sigma-Aldrich (St. Louis, MO, USA) and 1-methylanthracene (1-MeA; purity 99.5%) from Crescent Chemical (Islandia, NY, USA). All PAH stock solutions and forskolin (Sigma-Aldrich) and cyclic adenosine monophosphate (cAMP, Sigma-Aldrich) stock solutions were prepared in DMSO.

For the experiments performed in Germany, benzo[*a*]pyrene (99.9% purity) was purchased at Sigma-Aldrich (Taufkirchen, Germany), fluoranthene (98.6% purity) from AccuStandard (New Haven, CT, USA), and 1-methylanthracene (97.71% purity) from LGC Standards (Wesel, Germany). B[*a*]P-tetrol I-1 was purchased from Dr. Albrecht Seidel (BIU—Biochemical Institute for Environmental Carcinogens, Großhansdorf, Germany). The DNA adduct studies and cytochrome p450 studies were all conducted in Germany. All other studies were completed at the University of Colorado Anschutz.

### 2.2. Cell Culture

Immortalized human bronchial epithelial cells (BEAS-2B, ATCC, Manassas, VA, USA) of less than 10 passages were maintained at 37 °C and 5% CO_2_ with standard aseptic procedures. They were cultured in Dulbecco’s Modified Eagle Medium (DMEM; Gibco, Thermo Fisher Scientific, Waltham, MA, USA) media supplemented with 10% (*v*/*v*) fetal bovine serum (FBS) (Sigma-Aldrich) and 1% (*v*/*v*) antibiotic–antimycotic (Invitrogen). The BEAS-2B cells are commonly used for environmental toxicology studies, including our previous studies using nanotubes [38,39] and others, such as cigarette smoke (CS)-induced and environmental toxicant models of airway disease [40,41]. The BEAS-2B is a cell type that encounters exposures to toxicants preceding alveolar cells due to their location in the upper airways and can potentially undergo toxicant-induced cellular transformation [42].

The C10 cell line used for the micronuclei studies was obtained from Dr. Lori Nield (University of Colorado). C10 cells are an immortalized, non-transformed alveolar type II cell line originally derived from a BALB mouse [43]. C10 cells exhibit normal gap junctional intercellular communication and are well characterized by our laboratory [21,22,43]. Cells were used at <20 passage and maintained in CMRL 1066 media (Gibco)with 10% FBS and 1% glutamine in the same humidified atmosphere as BEAS-2B cells. The C10 cells were used for the micronuclei studies as a comparison to BEAS-2B cells and previous studies conducted with the C10 cells [20].

The A549 cells used for the *anti*-BPDE-DNA adduct analyses and cytochrome p450 1B1 immunoblots were obtained from CLS Cell Lines Service (Eppelheim, Germany) and are a tumorigenic lung adenocarcinoma cell line that is similar to alveolar type II cells and is used as a model for human alveolar type II cells [44,45]. These cells (passage <25) were maintained in DMEM/F12 media with GlutaMAX (Thermo Fisher, Darmstadt, Germany), 10% (*v*/*v*) FBS, and 1% (*v*/*v*) penicillin/streptomycin (Sigma-Aldrich, Taufkirchen, Germany) in the same humidified atmosphere as noted above.

Cells were grown to confluence (2–3 days), unless otherwise stated, in tissue culture dishes (Greiner, Cell Star, USA Scientific, Ocala, FL, USA). Once cells were confluent, they were treated with B[*a*]P, the 1:1 binary PAH mixture of 1-MeA and Flthn, or the combination of B[*a*]P + binary PAH mixture, as described in previous studies [20,22]. DMSO concentrations (<0.3%) did not elicit cytotoxicity to the cells; no differences between the DMSO and media control were observed in any of the experiments. All PAH doses used for the following endpoints were neither cytotoxic (Figure S1 for BEAS-2B cells and Figure S2 for A549 cells) nor elicited apoptotic or necrotic cell death (Table S1, BEAS-2B cells). Since no cytotoxicity was observed with the applied PAH concentrations in any of the cell lines used, the dose response experiments with the BEAS-2B and A549 for the DNA adduct studies were conducted over a broad concentration range. We determined the lowest doses with a response as well as the linear dose response range to choose suitable concentrations to study the mixture effects. These were the doses used for the remainder of the endpoints examined for the BEAS-2B and A549 cells for consistency. These doses were also used in previous studies as well as in the literature [5,20,46,47].

### 2.3. Micronucleus Assay

BEAS-2B and C10 cells were grown until 70% confluence on glass coverslips and treated with either B[*a*]P (0.1 or 0.3 µM), LMW PAH mix (0.01, 0.1 or 1.0 μM), or a combination of each dose of B[*a*]P and each dose of LMW PAH mix for 24 h. One similar dose of B[*a*]P was used for C10 cells (used previously, see [20]) to compare to BEAS-2B. DMSO served as a vehicle control. Following treatment, the coverslips were washed with PBS to remove the PAHs. The cells were fixed with ice cold methanol for 30 min and then mounted onto microscope slides using mounting medium containing DAPI (Vector, Burlingame, CA) to observe nuclear content. The cells were imaged using an Eclipse Ti-S microscope at 100× with a DS-QiMc camera (Nikon). Photographs were taken of at least 100 cells per slide and the number of micronuclei present was recorded. Three independent experiments were conducted for each cell type (BEAS-2B and C10).

### 2.4. Cell Cycle Analysis

BEAS-2B were grown to 70% confluence in 60 mm dishes (Greiner) and exposed to B[*a*]P (0.1 or 0.3 μM), LMW PAH mix (0.01, 0.1, or 1 μM), or each dose of B[*a*]P + LMW PAH mix for 24 h. After washing twice with PBS, cells were trypsinized and fixed in ethanol (Sigma-Aldrich). Kristnen stain containing propidium iodide (Sigma-Aldrich) was used to stain nuclear content and samples were analyzed using a BD Accuri C6 flow cytometer (BD Biosciences, San Jose, CA, USA). Ten thousand events were collected, and gating was set to exclude debris, non-cellular material, and doublets. Each experimental treatment with PAHs was conducted in triplicate and each of the triplicates was analyzed three times by flow cytometry. The percentage of cells in G1, S, and G2 phases of the cell cycle were determined via manual gating through FlowJo of the PI histogram and reported as a mean ± standard error of the mean (SEM) across all experiments (FlowJo v10, FlowJo, Ashland, OR, USA).

### 2.5. Scalpel-Loaded Dye Transfer Assay (SL/DT)

BEAS-2B cells were grown to confluence, serum-deprived for 24 h and concurrently treated with 1 µM cAMP and 5 µM forskolin to induce intercellular signaling as previously described [48], followed by exposure to either B[*a*]P (0.1 or 0.3 μM), LMW PAH mixture (0.01, 0.1 or 1.0 μM), or a combination of each dose of B[*a*]P + LMW PAH mixture. These doses were not cytotoxic in serum deprived cells (data not shown) nor in complete serum medium (Figure S1). As described previously [49], cells were washed with PBS three times, followed by scalpel loaded dye in three areas of each culture plate of cells in the presence of Lucifer yellow (1 mg/mL in PBS), and the dye was allowed to transfer through gap junctions for six minutes. Then, cells were fixed with 4% formalin (Sigma-Aldrich) and imaged using an Eclipse Ti-S microscope at 100× equipped with a DS-QiMc camera (Nikon Instruments, Melville, NY, USA). The area of dye spread in the image was quantified using ImageJ software (http://imagej.nih.gov/ij/, accessed on 15 January 2019) and analyzed by comparing either B[*a*]P, LMW PAH mix, or B[*a*]P + LMW PAH mix treated cells to DMSO control for the final fraction of control (FOC) percentages.

### 2.6. Experiments for DNA Adducts and Immunoblots

BEAS-2B cells were grown to confluence in 150 cm² and A549 cells were grown to at least 50% confluence in 75-cm² culture flasks (TPP, Trasadingen, Switzerland) prior to a 24 h treatment with the individual PAHs (B[*a*]P, Flthn, and 1-MeA) or B[*a*]P in combination with LMW PAHs. Test substances were first dissolved in DMSO and then further diluted in culture medium containing all supplements. The DMSO concentration in culture medium never exceeded 0.3% (*v*/*v*). These two cell types were chosen for these studies to represent upper airway and alveolar regions of the lung. After 24 h incubation, all test chemicals were removed, and cells were washed twice with PBS prior to detachment with trypsin-EDTA. Harvested cells were washed twice with PBS and centrifuged (250× *g*, 5 min, rt). After the last centrifugation step, the supernatant was discarded. Depending on the further use, cell pellets were either stored directly at −80 °C for adduct analyses or, for immunoblotting, were suspended in 300 µL RIPA buffer (0.1% *v*/*v* SDS (AppliChem, Darmstadt, Germany), 150 mM NaCl, 50 mM Tris-HCl, 1% *v*/*v* Nonidet™ P 40 Substitute (Sigma) and 0.5% *w*/*v* sodium deoxycholate (AppliChem) plus 1% *v*/*v* protease inhibitor cocktail (Sigma)). At least three independent experiments were conducted.

### 2.7. DNA Extraction and Quantitation

DNA was isolated from frozen cell pellets of PAH or DMSO treated cells (QIAamp DNA Blood Maxi Kit, Qiagen, Hilden, Germany) according to the manufacturer’s instructions. Nucleic acids were eluted in ultrapure water and the first two eluates were pooled. Afterwards, RNA was removed as follows: 10× concentrated TE buffer (100 mM Tris-HCl, 10 mM Na_2_-EDTA; pH 8.0), RNase A (final concentration 20 µg/mL; Qiagen) and RNase T_1_ (Sigma; final concentration 10 µg/mL) were added and samples were incubated for 30 min at 37 °C. Afterwards, 100 mM NaCl and 95% EtOH (2 volumes) were added. Samples were vigorously mixed and allowed to stand at room temperature for 10 min prior to centrifugation (13,000× *g*, 2 min, rt). Ultrapure water was used to dissolve the DNA pellet. Success of RNA removal was checked using Qubit^®^ dsDNA BR and Qubit^®^ RNA HS assay kits with a Qubit^®^ 3.0 fluorimeter (Thermo Fisher). Purified DNA was measured again on the day of sample preparation for chromatographic analysis. Then, DNA concentrations were determined with a NanoVue Plus^TM^ spectrophotometer (Biochrom Ltd., Cambridge, UK) and used for the calculation of DNA adduct rates.

### 2.8. Analysis of Anti-BPDE-DNA Adducts

Analysis of *anti*-BPDE-DNA adducts was carried out in terms of the B[*a*]P-specific analyte (±)-*r*-7,*t*-8,*t*-9,*c*-10-tetrahydroxy-7,8,9,10-tetrahydro-B[*a*]P (B[*a*]P-tetrol I-1) after acidic hydrolysis of DNA. The method was carried out as described previously by high-performance liquid chromatography with fluorescence detection (HPLC-FLD) [20] with the exception that a different analytical column was used (Gemini^®^ 5 µm NX-C18 110 Å, 250 × 4.6 mm, Phenomenex, Aschaffenburg, Germany). B[*a*]P-tetrol I-1 in the samples was quantified by comparison of the peak areas with those of the external calibration curve. Total B[*a*]P-tetrol I-1 mass and total DNA mass of each sample were calculated, and *anti*-BDPE-DNA adduct rates were assessed.

### 2.9. CYP1B1 Immunoblots

Frozen samples of treated cells (in RIPA buffer) were thawed, homogenized and centrifuged for 20 min (14,200× *g*, 4 °C). The supernatant was used for further analyses. The protein content of the samples was determined using the Pierce BCA Protein Assay Kit (Thermo Fisher) following the manufacturer’s instructions. Protein samples were further processed using the NuPAGE Bis-Tris electrophoresis system (Thermo Fisher). Gel electrophoresis and protein transfer were carried out as described previously with the following changes [47]. Per lane, 50 µg cell lysate of A549 or 10 µg of BEAS-2B or 7.8–62.5 µL CYP1B1 Corning^®^ Supersomes^TM^ (Corning, Amsterdam, The Netherlands) were applied. For the specific detection of CYP1B1 induction, a monoclonal mouse anti-CYP1B1 [G-4] antibody (4 µg/mL; Santa Cruz Biotechnology, Heidelberg, Germany) was used. Levels of CYP1B1 protein were evaluated using LabImage 1D L340 software (Intas, Göttingen, Germany). Relative CYP1B1 induction in treated cells was calculated in relation to the signals obtained for CYP1B1 microsomes, which served as reference standard.

### 2.10. Statistical Analysis

Statistical analysis was conducted using GraphPad Prism (La Jolla, CA, USA); statistical significance was set at *p* < 0.05. Group mean ± SEM are presented for all data. ANOVA was used for MN, cell cycle, gap junction, and cytotoxicity analyses followed by Tukey’s test for a posteriori comparison of means. For the *anti*-BPDE-DNA adduct analysis, *t*-tests were conducted to compare combinations of B[*a*]P and PAH mixture to B[*a*]P.

## 3. Results

### 3.1. Micronuclei Formation in Response to PAHs

**Figure 1:**
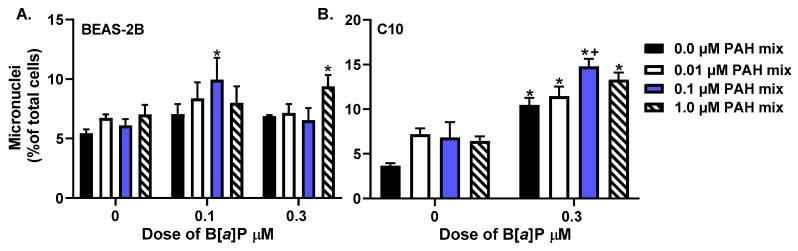
**Micronuclei formation in human and mouse lung epithelial cells exposed to PAHs** in (**A**) human BEAS-2B cells and (**B**) mouse C10 cells exposed to 0.01, 0.1, or 1 µM of the LMW PAH mixture (1:1, Flthn:1-MeA) and either 0.1 µM B[a]P and/or 0.3 µM B[a]P alone or in combination with the LMW PAH mixture for 24 h. Bars represent the mean percentage of micronuclei in the total number of cells analyzed in that treatment ± SEM; n = 3 samples, repeated 3 times; * significantly different from control, B[*a*]P and LMW PAH mixtures alone; + *p* < 0.05, significantly different from the B[*a*]P treatment alone.

BEAS-2B were analyzed for micronuclei formation, an indication of mutagenicity, following exposure to B[*a*]P (0.1 or 0.3 μM), the LMW PAH mixture (0.01, 0.1, or 1 μM), or each dose of the B[*a*]P + LMW PAH mixture for 24 h (Figure 1A). Cells treated with either the 0.1 μM B[*a*]P + 0.1 μM LMW PAH mixture or the 0.3 μM B[*a*]P + 1 μM LMW PAH mixture had significantly higher micronuclei formation as compared to the control, B[*a*]P, or the respective doses of the LMW PAH mixture. Additionally, we used C10 mouse alveolar type II cells to compare and validate the response in another non-transformed lung epithelial cell line used in our previous studies and observed significant increases above control with B[*a*]P alone and all three B[*a*]P + LMW PAH mixtures (Figure 1B). However, the B[*a*]P + 0.1 μM LMW PAH mixture was significantly elevated above that observed with the B[*a*]P or 0.1 μM LMW PAH mixture alone. For BEAS-2B, the control levels of micronuclei were similar to those previously observed [50,51]. These results provide evidence to support the genotoxic effects of B[*a*]P and these overlooked LMW PAHs in lung epithelial cells.

### 3.2. Anti-BPDE-DNA Adduct Formation in Response to PAHs

**Figure 2:**
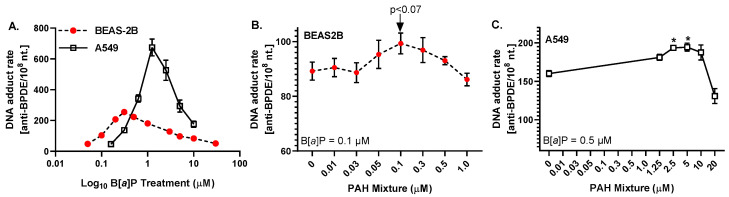
**Formation of *anti*-BPDE DNA adducts** in response to B[*a*]P in BEAS-2B and A549 cells (**A**), and to B[*a*]P in combination with increasing doses of LMW PAHs in BEAS-2B (**B**) and A549 cells (**C**). The data show mean ± SEM (n = 3); * *p* < 0.05 for 0.5 µM B[*a*]P combined with 2.5 or 5 µM LMW PAHs compared to B[*a*]P alone.

For comparison to our previous *anti*-BPDE-DNA adduct studies in the C10 lung epithelial cells [20], we exposed BEAS-2B and A549 cells to both B[*a*]P and the LMW PAH mixture. We evaluated dose responses for B[*a*]P after a 24 h incubation with B[*a*]P in both cell lines (Figure 2A) and observed a significant and dose-dependent increase in *anti*-BPDE-DNA adducts in both cell lines. Cell type differences (BEAS-2B, bronchial epithelial; A549, type II epithelial, tumorigenic) were evident and not unexpected. The starting point for *anti*-BPDE-DNA adduct formation between both cell lines was also similar, that is, the number of adducts at the lowest doses applied in our experiments (49 ± 14/10^8^ at 0.05 µM and 46 ± 18/10^8^ nucleotides at 0.16 µM B[*a*]P in BEAS-2B and A549, respectively). However, the maximum of adduct rates was detected in BEAS-2B cells (255 ± 36/10^8^ nucleotides) at much lower exposures (at 0.3 µM) compared to A549 cells, where the maximum of the curve (674 ± 95/10^8^ nucleotides) was observed at 1.25 µM (Figure 2A). On the other hand, in the low concentration range (0.05–0.3 µM), higher *anti*-BPDE-DNA adduct rates were observed in the BEAS-2B cells. The results show, expectedly, that BEAS-2B, a non-tumorigenic cell line, are much more sensitive to DNA adduct formation at lower concentrations compared to A549, a tumorigenic cell line. However, at the same time, BEAS-2B also show a response at much lower B[*a*]P doses to counteract the increasing adduct formation that otherwise might cause detrimental effects at the cellular level.

From the obtained dose responses, we determined the optimal dose of B[*a*]P to use for the combination studies with the binary mixtures of LMW PAHs. Therefore, the optimal dose of B[*a*]P was 0.1 μM for the BEAS-2B and 0.5 μM for the A549 cells to achieve ~40% of the maximum *anti*-BPDE-DNA adduct rates and thus being able to observe a further increase in DNA adduct formation in the presence of LMW PAHs.

Following a 24 h exposure to 0.1 μM B[*a*]P with increasing doses of LMW PAHs (Figure 2B), there were no significant increases observed in *anti*-BPDE DNA adducts in the BEAS-2B cells. As expected, the PAH mixture alone did not result in any detectable signals (Figure S3). However, a trend of increasing adduct levels (*p* < 0.07) was noted in the 0.1 μM B[*a*]P + 0.1 μM PAH mixture, corresponding to one of the same doses that elicits increased numbers of micronuclei (see Figure 1A). Significant increases in *anti*-BPDE-DNA adducts were observed in the A549 cells (Figure 2C) following 24 h of exposure to the 0.5 μM B[*a*]P + 2.5 and 5 μM PAH mixture. While these adduct levels are higher than those in BEAS-2B cells, they were similar to those previously observed in C10 cells, another type II cell line [20].

### 3.3. Cell Cycle Analysis of PAH-Treated Lung Epithelial Cells

Flow cytometry analysis of fluorescently labeled DNA in BEAS-2B cells exposed to B[*a*]P (0.1 or 0.3 μM), LMW PAH mix (0.01, 0.1, or 1 μM), or each dose of B[*a*]P + LMW PAH mix for 24 h indicated genotoxicity in terms of increased cell cycle arrest and supported the above-mentioned results on increased micronuclei formation.

**Figure 3:**
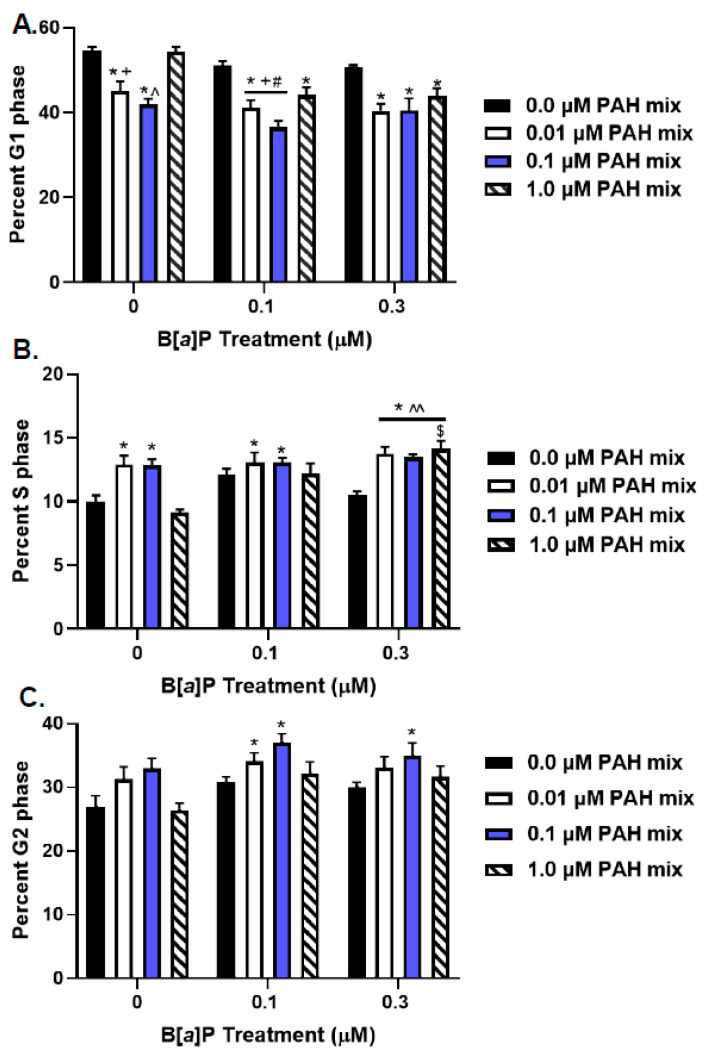
**Cell cycle changes observed in BEAS-2B cells exposed to PAHs.** BEAS-2B cells were exposed to 0.1 or 0.3 µM B[*a*]P alone; 0.01, 0.1, or 1 μM LMW PAH mix alone; a combination of the B[*a*]P and LMW PAH mix; or control (DMSO). Data represent mean percent of labeled DNA ± SEM in each phase of the cell cycle (G1 (**A**), S (**B**), and G2 (**C**); n = 3); * *p* < 0.05 significantly different from control; + *p* > 0.05 significantly different from 0.1 B[*a*]P; ^ *p* < 0.05 significantly different from either 0.1 or 0.3 B[*a*]P; # *p* > 0.05 significantly different from PAH mix combinations. ^^ *p* < 0.05 significantly different from 0.3 B[*a*]P; $ *p* < 0.05 significantly different from 1.0 PAH mix combination.

After 24 h, the BEAS-2B control cell cycle population demonstrated 54.6 ± 0.8, 10.0 ± 0.4, and 27.0. ± 1.7% of cells in the G1, S, and G2 phase, respectively. The S-phase cell populations after exposure to 0.1 and 0.3 μM B[*a*]P alone (12.1 ± 0.4 and 10.5 ± 0.3%, respectively) were not significantly different from those of the controls, whereas they were significantly elevated compared to the controls after exposure to the 0.01 and 0.10 μM LMW PAH mix alone (12.9 ± 0.7 and 12.8 ± 0.5%, respectively) (*p* < 0.05). In combined treatments with the 0.1 μM B[*a*]P + 0.01 or 0.1 μM LMW PAH mixture, no additional changes were observed (13.1 ± 0.8 and 13.1 ± 0.3). However, the S-phase populations after exposure to 0.3 μM B[*a*]P with all three combinations of LMW PAHs (0.01, 0.1, and 1 μM) were significantly elevated above both, in controls and after B[*a*]P treatment alone (Figure 3B; 13.8 ± 0.5, 13.5 ± 0.2, and 14.1 ± 0.6, respectively). Lastly, the 0.3 μM B[*a*]P + 1 μM LMW PAH mixture was also significantly elevated above that observed with 1 μM LMW PAH mixture alone. Thus, the combined effects of the 0.3 μM B[*a*]P + 1 μM LMW PAH mixture indicate that this combination has an increased and potentially additive effect regarding DNA synthesis.

There was also a significant difference in the percentage of cells exposed to either 0.1 μM B[a]P + 0.01 or 0.1 μM LMW PAH mixtures in the G2 phase compared to control; both were increased over control (Figure 3C). The effect from the 0.1 μM B[a]P + 0.1 μM LMW PAH mix was also significantly different from 0.1 μM B[*a*]P alone. Likewise, G2 phase cells treated with the 0.3 μM B[*a*]P + 0.1 μM LMW PAH mixture was also significantly elevated above control. A significant reduction in the percentage of cells in the G1 phase compared to the control was observed in cells exposed to the 0.01 or 0.1 μM LMW PAH mix alone and in combination with either B[*a*]P concentrations (Figure 3A). Additionally, G1 phase cells exposed to the 0.1 μM B[*a*]P + either 0.01 or 0.1 μM LMW PAH mixture were significantly reduced compared to either the 0.1 μM B[*a*]P or the LMW PAH mixtures at either dose alone. These data indicate that exposure to the B[*a*]P + LMW PAH mixture in the BEAS-2B cells resulted in cell cycle arrest, specifically increased S- and G2-phase and decreased G1-phase. These effects, if present, were increased and potentially additive. Twelve-hour treatments of BEAS-2B cells did not reveal any changes in S-phase, but did induce decreases in G1-phase cells and increases in G2 phase cells in combination treatments (Table S2).

Lastly, we used another human lung epithelial cell line (HBE1 cells) to further validate the observations in BEAS-2B cells (Table S3) that we recently published in additional PAH studies [52]. In these cells, both B[*a*]P concentrations led to increases in S-phase cells and decreases in G1 phase cells compared to controls; however, no changes were observed in the LMW PAH mixture treated cells. All combinations of B[*a*]P and LMW PAH mixtures resulted in similar findings with decreased G1-phase and increased S-phase cells with the exception of the 0.3 μM B[*a*]P + 0.1 μM LMW PAH mixture, where we also observed a significant increase compared to 0.3 μM B[*a*]P alone. Collectively, these results provide further evidence of increased and potentially additive cell cycle effects of PAH combinations between B[*a*]P and LMW-PAHs at non-cytotoxic doses.

### 3.4. GJIC in Response to PAHs

**Figure 4:**
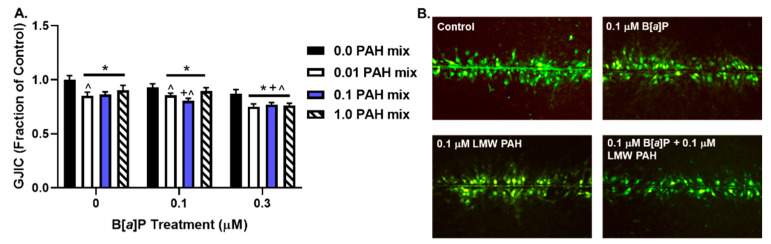
**GJIC dysregulation in BEAS-2B cells exposed to PAHs.** GJIC in BEAS-2B cells exposed to 0.01, 0.1, or 1 µM of the LMW PAHs and either 0.1 µM B[*a*]P or 0.3 µM B[*a*]P alone or in combination with the LMW PAHs. (**A**) Bars represent the mean ± SEM, as a percentage of GJIC exposed to control (DMSO); (n = 3) per treatment; repeated three times * *p* < 0.05, significantly different from control; + *p* < 0.05, significantly different from LMW PAHs; ^ significantly different from B[*a*]P. (**B**) Representative images of GJIC dysfunction in response to either control (DMSO), 0.1 µM B[a]P, 1 µM LMW PAHs, or a combination thereof.

BEAS-2B cells were analyzed for dysregulation of GJIC via the SL/DT assay following exposure to B[*a*]P (0.1 or 0.3 μM), LMW PAH mix (0.01, 0.1, or 1 μM), or each dose of B[*a*]P + LMW PAH mix for 24 h (Figure 4). All treatments induced significant inhibition in GJIC compared to the vehicle control. However, the 0.1 μM B[*a*]P + 0.1 μM LMW PAH mix and 0.3 μM B[*a*]P combined with each dose of the LMW PAH mix also produced a significant GJIC inhibition compared to either compound alone. This result indicates that the combination of B[*a*]P with the LMW PAH mix yields increased and potentially additive effects.

### 3.5. Cytochrome p450 1B1 Protein Expression in Response to LMW PAHs

Since CYP1A1 was neither detectable in BEAS-2B nor in A549 cells in immunoblots, CYP1B1 was studied instead and found to be induced upon 24 h B[*a*]P treatment (Figure S4). Overall, the effects were more pronounced in A549 than in BEAS-2B cells, i.e., larger differences between B[*a*]P and solvent treated cells were observed. Thus, we studied the effects of single and co-exposures with A549 cells only.

A steep and linear increase in the amount of CYP1B1 protein was observed up to 1.25 µM B[*a*]P. At higher concentrations, CYP1B1 induction continued to increase, but with a smaller slope (data not shown). Since CYP1B1 is involved in the metabolic activation of B[*a*]P and the increase in CYP1B1 induction fits well with the observed dose-dependent increase in *anti*-BPDE-DNA adducts (Figure 2A), we also chose 0.5 µM B[*a*]P as constant concentration for the combination studies with LMW PAHs. Since the effects of the LMW PAHs on CYP1B1 induction were unknown, we assessed the individual substances and binary mixtures.

**Figure 5:**
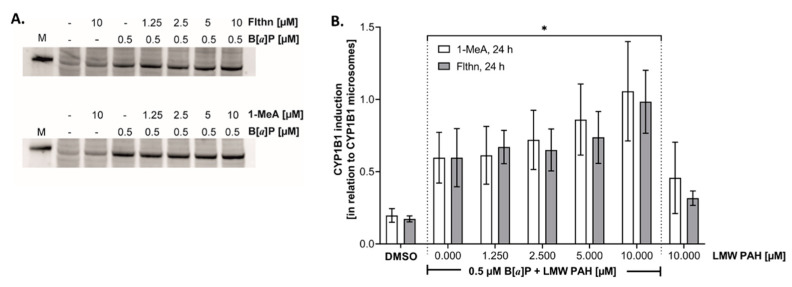
**Cytochrome p4501B1 immunoblots in A549 cells following PAH exposure.** (**A**) Immunoblot of A549 cells treated with B[*a*]P, 1-MeA, Flthn, or B[*a*]P combined with 1-MeA or Flthn for several concentrations. M = CYP1B1 microsomes (62.5 nL). (**B**) Graphic representation of the A549 immunoblots (n = 3 per samples repeated 3 times). * *p* < 0.05 compared to DMSO control.

Co-exposure of A549 cells with 0.5 µM B[*a*]P and increasing concentrations of both LMW PAHs, resulted in notable CYP1B1 induction (Figure 5A). The mean CYP1B1 induction of cells (relative to CYP1B1 induction in microsomes) treated with B[*a*]P alone was 0.60. With increasing exposures to 1-MeA, relative CYP1B1 induction increased up to 1.06 and with Flthn up to 0.98 (Figure 5B). This indicates that the combination of B[*a*]P with either one of the LMW PAH induces an additional effect for CYP1B1 induction.

## 4. Discussion

The data presented in this paper provide novel evidence of the co-mutagenic and co-carcinogenic effects of LMW PAHs (specifically the tested substances 1-MeA and Flthn) in human bronchial epithelial cell lines and a human alveolar type II cell line. The results further validate our previous research using different endpoints, species, and cell lines with the same PAH combinations, all at non-cytotoxic doses. Our prior data indicated that the tested combinations of B[*a*]P with the LMW PAHs 1-MeA and Flthn led to enhanced GJIC-dysregulation, increased *anti*-BPDE-DNA adducts, and an increase in an inflammatory mediator in C10 mouse lung cells [20]. In this paper, we show that, although species differences in responsiveness to these PAHs can occur, similar responses to these PAHs using several common endpoints (micronuclei, cell cycle, cell death, gap junction activity and *anti*-BPDE-DNA adducts) can be observed in human lung epithelial cell lines. The fact that the doses we used did not elicit cytotoxic nor apoptotic/necrotic events highlights the relevance of our results and supports the need for additional studies to better understand how LMW PAHs lead to adverse health effects in humans. The doses tested were both environmentally and occupationally relevant to human exposures, as noted previously by the dermal exposures of asphalt workers [31]. These PAHs are typically tested as a single component and assume that if a single PAH is not found to be carcinogenic, mutagenic, or elicit any effects by itself, that it is innocuous. However, exposure to PAHs is almost always via mixtures whether in the air, soil, or water, thus further supporting our use of several PAHs in combination. These specific PAHs are present in mainstream, secondhand, and thirdhand cigarette smoke as well as found in marijuana smoke [11]; secondhand cigarette smoke levels reach >5.0 μg total PAH/cigarette in some brands [5,53,54,55,56]. The LMW PAH fraction of these mixtures accounts for ~70% of the total PAHs. These PAHs are also present in other environmental settings, such as detected in bivalve tissues from the Fjords in Canada [57] to soil sediments along rivers in the Czech Republic to China [30,58,59], to wildfires from California to Portugal [60,61], as just a few examples to further support the prevalence of these ubiquitous PAHs. This is not including those workers exposed occupationally, for which there are many, in occupations such as asphalt, coal, coke and steel industry workers [1,17,26,27,28,29,30].

In these studies, we evaluated mutagenicity of these PAHs using micronuclei formation and observed that the combination of the B[*a*]P and binary LMW PAHs (1-MeA and Flthn) significantly increased micronuclei levels above those that were observed after exposure to B[*a*]P or LMW PAHs alone (0.1 μM B[*a*]P combined with 0.1 μM PAH mixture). Similar increased and potentially additive results were observed here, in human lung epithelial cell lines, for the formation of *anti*-BPDE-DNA adducts. These effects are also in line with those observed in C10 cells where we previously observed an increase in *anti*-BPDE-DNA adducts in B[*a*]P and LMW PAHs-treated cells compared to B[*a*]P and LMW-PAHs alone [20]. Conversely, we used C10 cells in our studies in this paper to compare and validate the BEAS-2B micronuclei results and observed similar results in both cell lines. When comparing the BEAS-2B *anti*-BPDE-DNA adducts formed upon B[*a*]P exposure, the adduct levels are less than those observed in C10 cells. However, the *anti*-BPDE-DNA adduct response in the A549 cells is similar to the C10 cells. This difference may reflect the cell of origin, i.e., BEAS-2B cells are derived from non-tumorigenic bronchial epithelial cells from higher up in the bronchial tree versus the C10 (non-tumorigenic) and A549 (tumorigenic) cells that are derived from alveolar type II cells. Lastly, because both increased DNA adduct and micronuclei formation are important genotoxic and mutagenic events that contribute to the initiation of cancer, our results provide evidence of the co-carcinogenic potential of LMW PAHs in combination with B[*a*]P, a known carcinogen.

CYP1B1 plays a role in the metabolic activation HMW PAHs to DNA reactive intermediates and is inducible by ligands of the aryl hydrocarbon receptor (AhR), which acts as a transcription factor [62,63,64]. Though the present study did not directly analyze the enzyme activity of CYP1B1, the increased formation of *anti*-BPDE-DNA adducts in combination with increasing CYP1B1 on the protein level upon B[*a*]P exposure is indirect evidence of enzymatic activity. However, in A549 as well as in BEAS-2B cells, a dose-dependent increase in *anti*-BPDE-DNA adducts was only observed up to 1 and 0.3 µM B[*a*]P, respectively, and then followed by a continuous decrease (Figure 2A). Potential reasons for the observed decrease in DNA adducts while the amount of CYP1B1 protein still increases could be an induction of detoxifying enzymes, such as glutathione S-transferases, DNA repair, or—as shown in experiments with human CYP1B1 microsomes—a competitive inhibition of CYP1B1 activity by B[*a*]P or its metabolites such as diol [64,65,66]. In lung cells and other cellular models, non-tumorigenic cells are more sensitive relative to tumorigenic cells, which acquire characteristics that make them more resistant to stress factors and prone to grow uncontrolled [67,68,69]. For example, increased DNA repair is known to occur in tumorigenic cells that survive chemotherapy and radiation therapy [67]; in A549 cells compared the BEAS-2B cells, the DNA repair machinery for non-homologous end joining is increased in response to nickel exposure [69].

As mentioned earlier, the experiments with the LMW mixtures were carried out with constant B[*a*]P doses that were below the maximum DNA adduct rates where CYP1B1 protein and activity correlate. We observed a dose dependent, but not significant, increase in *anti*-BPDE-DNA adducts when BEAS-2B cells were co-exposed to B[*a*]P and the LMW PAH mixture. It is possible that this increase is directly linked to CYP1B1 activity, but it remains speculative. The effect of the PAH mixture on CYP1B1 induction was not further investigated in the present study as the A549 cells in response to Flthn or 1-MeA alone was very low and B[*a*]P combined with Flthn or 1-MeA were also not significantly different from B[*a*]P. Future studies will further evaluate metabolic differences with other and more sensitive methods. Cell cycle changes, particularly increases in S-phase and cell cycle arrests, are indicative of several of the hallmarks of cancer [68] and are also common in early stage cancer development. Although the changes we observed were small, they were significant and correlated to the observed increases in genotoxicity (i.e., micronuclei). Specifically, cell cycle arrest in G1/S was concurrent to increases in genotoxicity and mutagenicity; significant G1 arrest was observed specifically in response to B[*a*]P combined with LMW PAHs (Figure 3A). These types of G1/S arrests support a potential interaction of the studied PAHs with the centrosome, among other damage [38]. We also evaluated HBE1, another human lung epithelial cell line, for PAH-induced cell cycle changes for validation (Table S3) and observed increased cell cycle arrests after exposure to mixtures of B[*a*]P and LMW PAHs compared to single PAH exposures.

One common endpoint between the studies in murine [19] and the human lung cells described in this paper was the assessment of gap junction activity (GJIC) that also resulted in similar observations between C10 and BEAS-2B cells. As noted earlier, the inhibition of gap junctions is a well-known feature of evading growth suppression, a hallmark of cancer, and therefore is also involved in the early events of cancer development. There are numerous papers from our laboratories and others that indicate inhibition of GJIC occurs in response to LMW PAHs in human and mouse lung epithelial cells, rat liver cells, and human pancreatic cells [21,22,48,52,70,71]. The current findings support the use of GJIC as a valid endpoint for detecting whether PAHs have an adverse effect. For example, we noted that 0.1 μM B[*a*]P, a PAH that is typically set at 1 for toxic equivalency factors, has 6.9% reduced GJIC compared to control cells, whereas at the same dose (0.1 μM) of the LMW PAH mixture, there is a 13.7% reduction compared to the control cells, equating to almost a two-fold difference in responsiveness. We also noted that, when comparing these findings to the combination of B[*a*]P and a 1:1 mixture of LMW PAH at 0.1 μM (total [PAH] is 0.2 μM), the response is almost additive, with a 19.6% reduction. These data provide further evidence that additional endpoints, such as GJIC, should be evaluated when determining the potential of a compound to contribute to cancer development rather than the “classical” genotoxic and mutagenic endpoints only, such as micronuclei or DNA adduct formation. Further, our data generally support the importance of assessing PAH mixtures, including high-abundant LMW PAHs, such as methylanthracenes, rather than single PAHs only.

## 5. Conclusions

Our novel studies in human lung epithelial cells support the potential role of PAHs in eliciting adverse health effects in humans, particularly co-carcinogenic effects. While B[*a*]P is a well-known carcinogen, the other two PAHs (1-MeA and Flthn) are much less studied and not regarded as human carcinogens. In this paper, we show that non-cytotoxic doses of these PAHs additionally increase B[*a*]P-induced micronuclei, *anti*-BPDE-DNA adduct formation, S phase of the cell cycle, and, even in the absence of B[*a*]P, reduce gap junction activity that is indicative of early stages in cancer development. Collectively, our data overall support the hypothesis that LMW PAHs act as co-carcinogens with B[*a*]P and provide valuable insight into additional endpoints, such as cell cycle arrest and GJIC dysregulation, which should be used in the future to initiate more risk assessment studies for PAHs. Future studies will address the downstream regulation of these events, such as understanding how the cell cycle is arrested (e.g., through p53 and/or p21 mechanisms), the use of a pan-centromeric probe [72] that will allow for the identification of potential mitotic disruption and errors in chromosome number, understanding any changes in DNA repair, and understanding the events downstream of GJIC dysregulation, such as an imbalance in bioactive lipids (i.e., eicosanoids) leading to potential adverse effects (e.g., cancer and inflammation) [23]. These endpoints, linking PAHs to adverse lung effects, are also critical to assess potentially upcoming risks, such as the increasing numbers of wildfires based on global warming, and, therefore, increased air pollution and PAH exposure [73,74].

## Data Availability

Not applicable.

## Supplementary Materials

The following are available online at https://www.mdpi.com/article/10.3390/toxics10010028/s1: Supplementary Materials and Methods: Table S1: Significant cell death was not observed in response to exposure to B[*a*]P and the LMW PAH mixture for 24 h in BEAS-2B cells; Table S2: Significant cell cycle changes in BEAS-2B cells were observed 12 h following B[*a*]P and the LMW PAH mixture treatment; Table S3: Significant cell cycle changes in HBE1 cells were observed 24 h following B[*a*]P and the LMW PAH mixture treatment; Figure S1: BEAS-2B viability after 24 or 48 h exposure to PAH combinations; Figure S2: Neutral red uptake indicative of cytotoxicity in A549 cells after 24 h incubation with individual PAHs, B[*a*]P combined with the individual LMW PAHs (1-MeA or Flthn), or the PAH mixture (Flthn:1MeA; 1:1 ratio); Figure S3: Representative chromatograms from HPLC analysis of B[*a*]P-tetrol I-1; Figure S4: Cytochrome p450 1A1 and 1B1 in immunoblots in BEAS-2B an A549 cells.
