# Supplementary material for: The Carcinogenic Properties of Overlooked yet Prevalent Polycyclic Aromatic Hydrocarbons in Human Lung Epithelial Cells
Source: Toxics. 2022 Jan 9;10(1):28. doi: 10.3390/toxics10010028 (PMC8779510; doi:10.3390/toxics10010028)
Supplement: Supplementary file 1 [file toxics-10-00028-s001.zip › toxics-1506318-supplementary.pdf]

toxics

Supplementary Materials: The Carcinogenic Properties of Overlooked yet Prevalent Polycyclic Aromatic Hydrocarbons in Human Lung Epithelial Cells

Alison K. Bauer, Katelyn J. Siegrist, Melanie Wolff, Lindsey Nield, Thomas Brüning, Brad L. Upham, Heiko U. Käfferlein, Sabine Plöttner

Table S1. Significant cell death was not observed in response to exposure to B[*a*]P and the LMW PAH mixture for 24 h.

Treatment (mM)	Apoptosis (Mean Percent Control ± SEM)	Apoptosis *p* Value	Necrosis (Mean Percent Control ± SEM)	Necrosis *p* Value
Control	100 ± 10.32		100 ± 40.46	
Camptothecin	190.66 ± 73.45	*	526.37 + 471.47	*
H_2O_2	234.76 ± 65.15	*	510.55 ± 136.11	*
0.10 B[*a*]P	124.83 ± 21.85	ns	214.53 ± 59.79	ns
0.30 B[*a*]P	92.42 ± 2.4	ns	173.1 ± 66.87	ns
0.01 PAH mix	99.28 ± 16.14	ns	212.61 ± 58.58	ns
0.10 PAH mix	71.62 ± 6.58	ns	142.37 ± 50.97	ns
1.0 PAH mix	81.19 ± 6.11	ns	133.89 ± 46.14	ns
0.10 B[*a*]P + 0.01 PAH mix	92 ± 13.06	ns	285.58 ± 60.91	ns
0.10 B[*a*]P + 0.10 PAH mix	97.36 ± 11.02	ns	242.23 ± 61.84	ns
0.10 B[*a*]P + 1.0 PAH mix	114.05 ± 13.21	ns	324.48 ± 38.54	ns
0.30 B[*a*]P + 0.01 PAH mix	84.36 ± 13.93	ns	274.84 ± 58.87	ns
0.30 B[*a*]P + 0.10 PAH mix	117.18 ± 7.66	ns	418.17 ± 50.73	*
0.30 B[*a*]P + 1.0 PAH mix	92.90 ± 10.13	ns	265.09 ± 59.94	ns

* $p < 0.05$ compared to control.

Table S2. Significant cell cycle changes in BEAS-2B cells were observed 12 h following B[*a*]P and the LMW PAH mixture treatment.

Treatment (µM)	G1 Phase (Mean±SEM)	S Phase (Mean±SEM)	G2 (Mean±SEM)
Control	43.6 ± 0.88	12.0 ± 1.28	40.1 ± 1.88
0.10 B[*a*]P	43.3 ± 0.83	12.5 ± 1.55	40.3 ± 1.59
0.30 B[*a*]P	46.1 ± 0.91	12.8 ± 1.19	37.7 ± 1.31
0.01 PAH mix	42.1 ± 1.24	10.8 ± 1.15	44.3* ± 1.75
0.10 PAH mix	42.0 ± 1.23	12.4 ± 1.75	43.3 ± 2.33
1.0 PAH mix	43.0 ± 0.96	12.7 ± 1.34	41.7* ± 1.57
0.10 B[*a*]P + 0.01 PAH mix	41.2 ± 1.92	12.7 ± 0.80	43.1 ± 2.45
0.10 B[*a*]P + 0.10 PAH mix	41.3 ± 1.33	12.9 ± 0.95	43.2 ± 1.84
0.10 B[*a*]P + 1.0 PAH mix	40.5* ± 2.4	10.7 ± 1.07	46.7* ± 2.72
0.30 B[*a*]P + 0.01 PAH mix	37.6* ± 2.29	12.8 ± 1.08	47.6* ± 3.17
0.30 B[*a*]P + 0.10 PAH mix	36.8* ± 2.62	13.8 ± 1.19	47.6* ± 3.6
0.30 B[*a*]P + 1.0 PAH mix	38.4* ± 1.61	13.3 ± 0.82	46.1* ± 2.34

*$p < 0.05$ significantly different from Control.

Table S3. Significant cell cycle changes in HBE1 cells were observed 24 h following B[*a*]P and the LMW PAH mixture treatment.

Treatment (μM)	G1 Phase (Mean±SEM)	S Phase (Mean±SEM)	G2 (Mean±SEM)
Control	72.5 ± 1.69	9.6 ± 1.06	16.0 ± 1.07
0.10 B[*a*]P	64.44* ± 2.99	14.8* ± 2.08	19.3 ± 1.42
0.30 B[*a*]P	66.8* ± 2.44	14.8* ± 2.38	17.0 ± 1.03
0.01 PAH mix	71.9 ± 1.53	10.7 ± 0.72	16.0 ± 1.45
0.10 PAH mix	70.1 ± 1.74	10.6 ± 1.32	18.2 ± 0.86
1.0 PAH mix	70.5 ±2.3	10.8 ± 1.17	17.7 ± 1.61
0.10 B[*a*]P + 0.01 PAH mix	67.21*± 3.85	14.09* ± 1.96	17.6 ± 2.52
0.10 B[*a*]P + 0.10 PAH mix	62.96*± 3.96	15.60* ± 2.24	20.6* ± 2.18
0.10 B[*a*]P + 1.0 PAH mix	68.1 ± 2.13	14.79* ± 1.94	16.1 ± 0.69
0.30 B[*a*]P + 0.01 PAH mix	65.58* ± 1.52	16.05* ± 2.19	16.1 ± 2.25
0.30 B[*a*]P + 0.10 PAH mix	62.59* ± 1.3	18.05*+ ± 2.69	17.9 ± 1.2
0.30 B[*a*]P + 1.0 PAH mix	64.7* ± 1.2	15.4* ± 1.73	18.3 ± 1.98

* Significantly different from control, $p < 0.03$; +Significantly different from B[a]P alone, $p < 0.01$

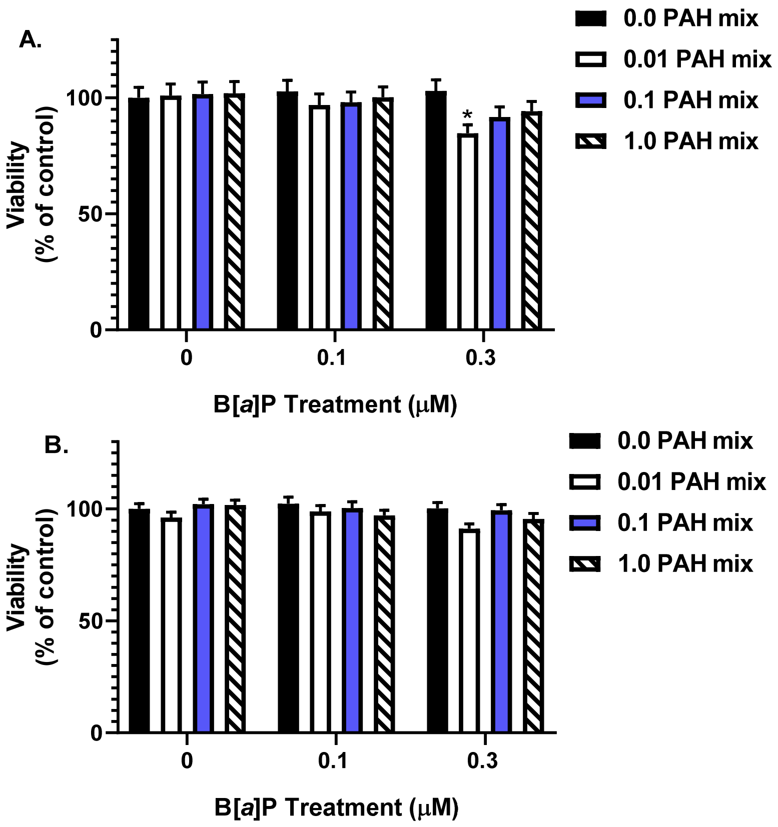

Figure S1. BEAS-2B viability after 24 or 48 h exposure to PAH combinations. Viability of BEAS-2B cells exposed to 0.01, 0.1, or 1 μM of the LMW PAH mixture (1:1, Flnth:1-MeA) and either 0.1 or 0.3 μM B[a]P alone or in combination with the LMW PAH mixture for 24 h (**A.**) or 48 h (**B.**). Bars represent the mean ± SEM, as a percentage of viable cells exposed to control (DMSO) in complete serum medium; n = 3 per treatment; repeated three times. * $p < 0.05$, significantly different from control.

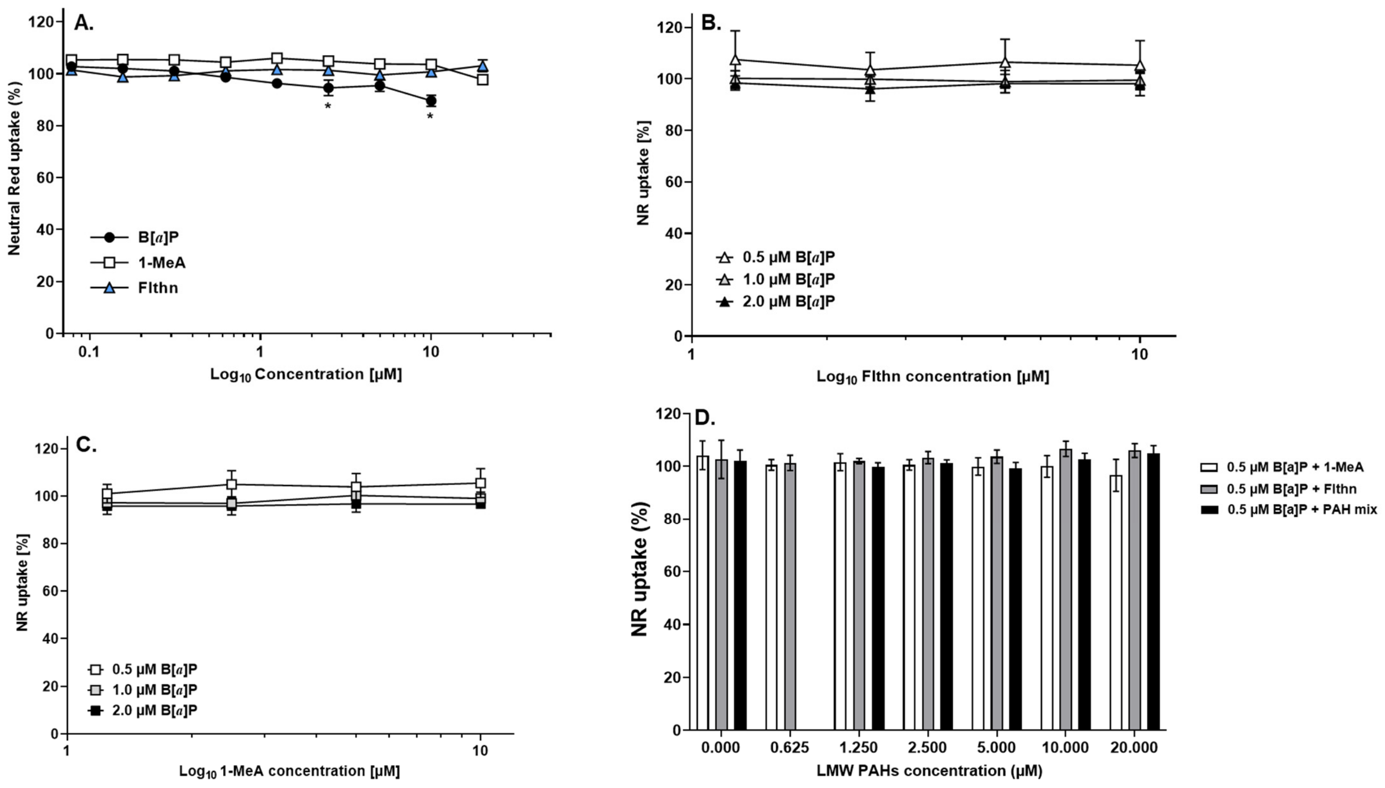

Figure S2. Neutral red uptake indicative of cytotoxicity in A549 cells after 24-h incubation with individual PAHs, B[*a*]P combined with the individual LMW PAHs (1-MeA or Flthn), or the PAH mixture (Flthn:1MeA; 1:1 ratio). A. Individual PAHs; **B**. B[*a*]P combined with Flthn; **C**. B[*a*]P combined with 1-MeA; **D**. 0.5 mM B[*a*]P combined with the individual LMW PAHs or combined with both LMW PAHs (PAH mixture). * $p < 0.05$ for these concentrations of B[*a*]P compared to no toxicity set at 100%.

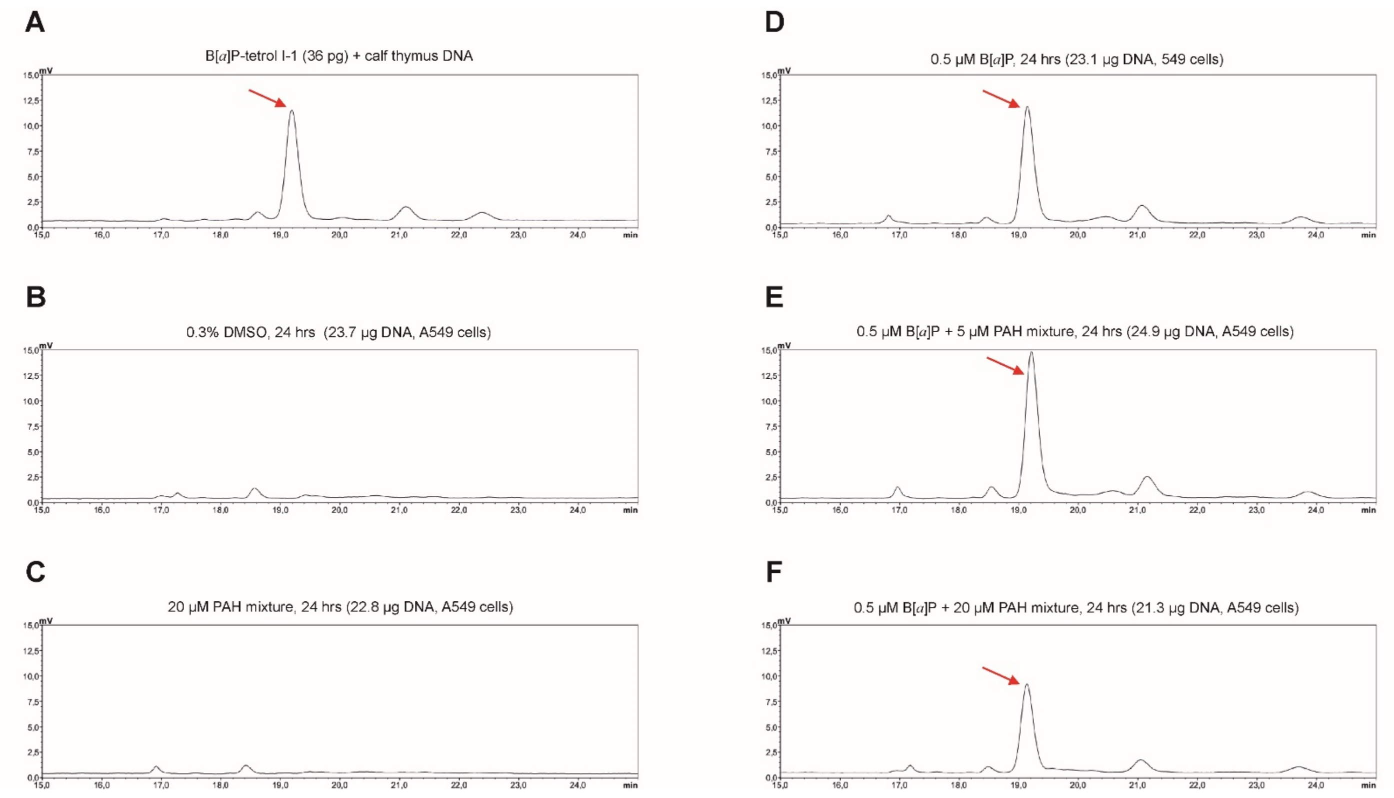

Figure S3. Representative chromatograms from HPLC analysis of B[*a*]P-tetrol I-1. A. Calf thymus DNA spiked with 36 pg B[*a*]P-tetrol standard; **B.-F**. Isolated DNA of A549 cells treated with **B.** DMSO; **C**. 20 µM PAH mixture; **D**. 0.5 µM B[*a*]P; **E**. 0.5 µM B[*a*]P + 5 µM PAH mixture; **F**. 0.5 µM B[*a*]P + 20µM PAH mixture. Red arrows point towards the peak of B[*a*]P-tetrol I-1.

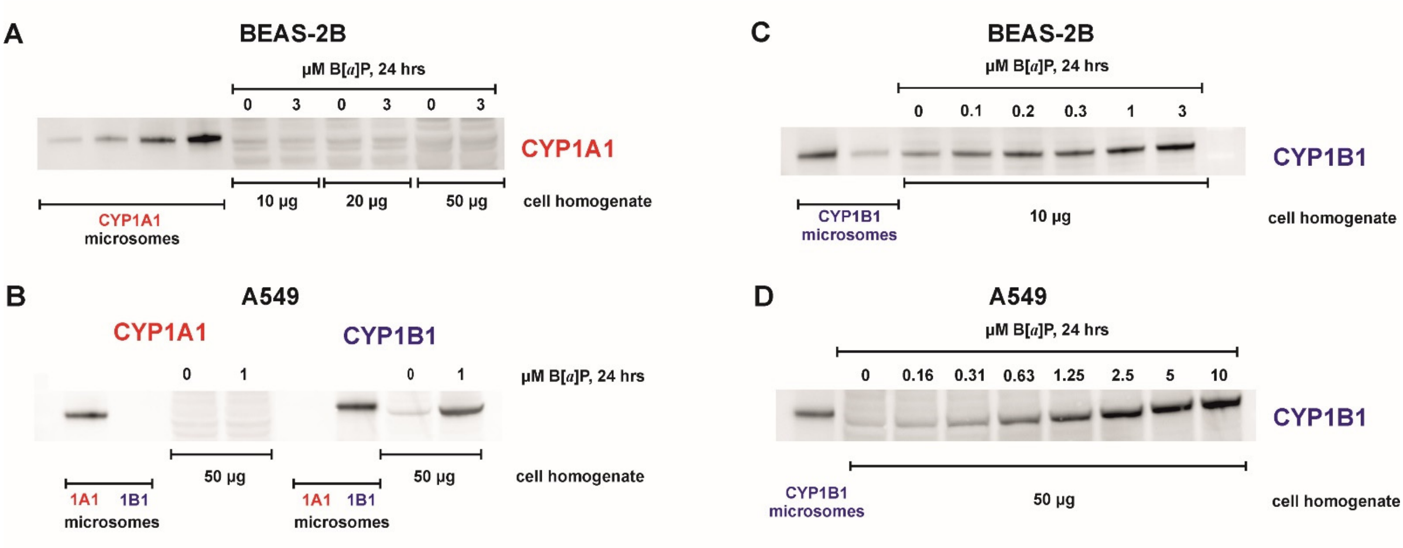

Figure S4. Cytochrome p450 1A1 and 1B1 in immunoblots in BEAS-2B an A549 cells. A. Absence of CYP1A1 in BEAS-2B cells after 24-h treatment with DMSO or 3 µM B[*a*]P. CYP1A1 microsomes (0.98, 1.95, 3.9 and 7.8 nL) or 0, 20 or 50 µg cell homogenate were applied. **B**. Absence of CYP1A1 in A549 cells after 24-h treatment with DMSO or 1 µM B[*a*]P (left panel) and presence of CYP1B1 in the same samples (right panel). No cross-reaction with the anti-CYP1A1 antibody and CYP1B1 microsomes (left) and anti-CYP1B1 antibody and CYP1A1 microsomes (right). 3.9 nL CYP1A1 or 62.5 nL CYP1B1 microsomes or 50 µg cell homogenate were applied. **C**. Dose-dependent induction of CYP1B1 in BEAS-2B cells upon 24-h exposure to 0.1-3 µM B[a]P. 31.3 and 7.8 nL CYP1B1 mircosomes or 10 µg cell homogenate were applied. **D**. Dose-dependent CYP1B1 induction in A549 cells after 24-h exposure to 0.16–10 µM B[*a*]P. 62.5 nL CYP1B1 mircosomes or 50 µg cell homogenate were applied.

Supplementary Materials and Methods

Cytotoxicity and apoptosis/necrosis analysis. Cytotoxicity assays were done with fully confluent BEAS-2B treated with either 0.1 or 0.3 µM B[a]P, 1, 0.1. or 0.01 µM LMW PAH mix, or a combination of each dose of B[a]P + LMW PAH mix for 24-48 hours in media with or without 10% serum. Cells were washed with PBS after treatment and CellTiter 96 AQueous One Solution Cell Viability assay (MTS assay, Promega) was performed as described by manufacturer. The toxicity observed was similar with or without serum. Mean values ± standard error (SE) were calculated from three independent experiments.

Cytotoxic effects of the PAHs in A459 cells were assessed utilizing the neutral red uptake assay following the protocol by Repetto et al. (2008) with some minor modifications. In brief, cells were seeded per well in 96-well microplates and cultured overnight. The following day, culture medium was replaced by medium containing either individual PAHs (0.039–10 µM B[a]P, 0.078–20 µM 1-MeA or 0.078–20 µM Flthn) or 0.5, 1 or 2 µM B[a]P in combination with 1.25–10 µM 1-MeA or 1.25–10 µM Flthn. 0.1% and 0.2% DMSO served as negative control. After 24-h, media were removed and replaced by media containing 50 µg/ml neutral red (NR). After 3-h, the supernatant was removed, and cells washed 3X with PBS/well using a HydroFlex™ microplate washer (Tecan, Crailsheim, Germany). After washing, 200 µl fixative (50% v/v ethanol, 1% v/v acetic acid, 49% v/v ultrapure water) were added and plates were shaken for 30 min at 580 rpm on a plate shaker. Absorbance of extracted NR was measured at 540 nm using an Infinite M200 microplate reader (Tecan). Mean absorbance values of the six replicates per treatment were calculated and blank values were subtracted. Neutral red uptake was calculated,

following the manufacturers protocol. Mean values ± standard deviation (SD) for neutral red uptake were calculated from three independent experiments.

Annexin-V/propidium iodide staining was used for detection of apoptosis and necrosis in the BEA2-2B cells. BEAS-2B cells were grown until 70% confluence and treated with either B[*a*]P (0.1 or 0.3 µM), LMW PAH mix (0.01, 0.1 or 1.0 µM), or a combination of each dose of B[*a*]P + each dose of LMW PAH mix for 24 hours. DMSO served as a vehicle control; 6 µM Camptothecin (Cayman Chemical) was used as a positive control for apoptosis and necrosis following a 6 hr treatment and 10 mM H_2O_2 (Sigma-Aldrich) as a positive control for necrosis and apoptosis after 1 hr treatment. The cells were then washed once with PBS followed by staining buffer (10 mM HEPES, 140 mM NaCl, 2.5 mM $CaCl_2$, pH 7.4). Treatment groups were then each incubated with 2.5 ul Annexin-V-APC (Molecular Probes, A35110), 200 µl propidium iodide containing buffer (3 µM), and 200 µl binding buffer for 15 min in the dark at room temperature. Cells were then analyzed using a BD Accuri C6 flow cytometer (BD Biosciences, San Jose, CA). Ten thousand events were collected and gating was set to exclude debris and non-cellular material. Each treatment was performed in triplicate and the experiment was repeated three times. The percentage of apoptotic and necrotic cells were determined via manual gating through FlowJo and reported as a mean ± SEM across all experiments (FlowJo v10, FlowJo, Ashland, OR, USA).

HBE1 cell maintenance and cell cycle analysis. Nontumorigenic immortalized human bronchial epithelial cell line HBE1 was kindly provided by Dr. Reen Wu (University of California Davis). HBE1 cell line originates from non-cystic fibrosis lung tissue of 60-year old woman(Yankaskas et al, 1993) Cells were grown in a serum-free, phenol red-containing DMEM/F12 (Invitrogen) supplemented with 4 µg/mL human insulin, 5 µg/mL transferrin, 10 ng/mL human EGF, 0.1 µM dexamethasone, 20 ng/mL cholera toxin, 2.5 µg/mL Plasmocin treatment, 2.5 mM L-glutamine and 0.1% (v/v) endothelial cell growth supplement. After adding all the supplements, pH was adjusted to 7.40 ± 0.05 and medium was sterile-filtered. Cells were routinely cultured and split at 70% confluence and used < passage 10. Cells were prepared in the same manner as the BEAS-2B for cell cycle analysis.

References

1. Repetto, G.; del Peso A., Zurita, J.L. Neutral red uptake assay for the estimation of cell viability/cytotoxicity. *Nat. Protoc.*, **2008**, *3*(7), 1125–1131.
2. Yankaskas, J.R.; Haizlip, J.E.; Conrad, M.; Koval, D.; Lazarowski, E.; Paradiso, A.M.; Rinehart Jr, C.A.; Sarkadi, B.; Schlegel, R.; Boucher, R.C. Papilloma-virus immortalized tracheal epithelial cells retail a well-differentiated phenotype. *Am. J. Physiol.*, **1993**, *264*, C1219–C1230.
